# Algorithmic fairness in pandemic forecasting: lessons from COVID-19

**DOI:** 10.1038/s41746-022-00602-z

**Published:** 2022-05-10

**Authors:** Thomas C. Tsai, Sercan Arik, Benjamin H. Jacobson, Jinsung Yoon, Nate Yoder, Dario Sava, Margaret Mitchell, Garth Graham, Tomas Pfister

**Affiliations:** 1 Department of Health Policy and Management, Harvard T.H. Chan School of Public Health, Boston, MA USA; 2 Department of Surgery, Brigham and Women’s Hospital, Boston, MA USA; 3 Google Inc., Mountain View, CA USA; 4 Present Address: Stanford University School of Medicine, Stanford, CA USA

**Keywords:** Epidemiology, Epidemiology

## Abstract

Racial and ethnic minorities have borne a particularly acute burden of the COVID-19 pandemic in the United States. There is a growing awareness from both researchers and public health leaders of the critical need to ensure fairness in forecast results. Without careful and deliberate bias mitigation, inequities embedded in data can be transferred to model predictions, perpetuating disparities, and exacerbating the disproportionate harms of the COVID-19 pandemic. These biases in data and forecasts can be viewed through both statistical and sociological lenses, and the challenges of both building hierarchical models with limited data availability and drawing on data that reflects structural inequities must be confronted. We present an outline of key modeling domains in which unfairness may be introduced and draw on our experience building and testing the Google-Harvard COVID-19 Public Forecasting model to illustrate these challenges and offer strategies to address them. While targeted toward pandemic forecasting, these domains of potentially biased modeling and concurrent approaches to pursuing fairness present important considerations for equitable machine-learning innovation.

## Introduction

In response to the 2019 novel coronavirus disease (COVID-19) pandemic, there has been significant investment in the development of forecasting models. The Centers for Disease Control and Prevention (CDC) COVID-19 Forecast hub incorporated forecasts on mortality from 23 models representing contributions from over 50 academic, industry, and independent research groups^1^. Both the CDC and the American Hospital Association (AHA) have called on hospitals and public health officials to rely on forecasting to help guide decision-making^2,3^. Given the complexity of both compartmental epidemiologic and machine-learned pandemic forecasting models, concern has been raised about the potential that inaccurate or unfair models could worsen healthcare disparities^4^. An unfair model may display differing levels of accuracy in making predictions for different subgroups or could rely on false correlations and associations, leading to downstream inequities if acted on. Though fair and effective forecasting models have the potential to inform the public on the likely course of the pandemic and to guide policymakers on the allocation of scarce resources, usage of unfair models could instead worsen disparities by failing to recognize the disproportionate burden experienced by minority communities facing poor access to testing, increased risk of exposure due to essential work, and other long-standing health inequities.

COVID-19 has had a disproportionate impact on racial/ethnic minority communities in the United States, and it is critical that policy actions recognize and address these inequities. There is long-standing literature on the persistence of disparities in health outcomes, and social determinants have been the primary drivers of these disparities^5–9^. While salient for COVID-19, these disparities in health outcomes are in part due to the consequences of structural inequities and existed long before the pandemic^10,11^. Wide variations in life expectancy persist across counties and cities, and the largest predictors of this disparity remain the effect of socioeconomic and minority status^12–14^. Black and Hispanic communities in the United States often face steep barriers to healthcare access^15^, but inequities are so ingrained into healthcare systems that substantial inequities in outcomes persist even after adjusting for disparities in healthcare access^16,17^. These same lower-income and minority communities have also borne the brunt of the COVID-19 pandemic^18^. Black, Hispanic/Latino, and Indigenous communities have seen much greater rates of COVID-19 than other communities in the United States, and they have had three to four times higher hospitalization rates than their White counterparts^19^. On top of these structural inequities, there is evidence that healthcare tools and algorithms themselves may worsen disparities and redirect clinical attention in ways that could worsen inequities^20–22^. Given the proliferation of COVID-19 forecasting models, the years of healthcare inequity caused by a history of structural racism in the US, and the encoding of inequity in many healthcare tools, policymakers should recognize the possibility of predictive algorithms perpetuating or exacerbating existing disparities if not deliberately developed to ensure equity. As we grow ever more aware of the ways that well-intentioned innovation can instead perpetuate structural disparities, we have both the opportunity and the obligation to do better.

In this manuscript, we outline key challenges to the fair and equitable development of pandemic forecasting models from both a statistical and social epidemiology perspective. Using our experiences building the Google-Harvard COVID-19 Public Forecasting model as an example, we suggest ways to resolve these barriers and to help ensure fairness. Our approach has been informed by the robust literature demonstrating the potential for health interventions to actually worsen disparities if not undertaken deliberately^23–25^, and with a careful understanding of inequities, structural racism, and the need to build models that actively work toward increasing equity^26–30^. Therefore, our conceptual framework begins with an explicit focus on equity and fairness instead of simply focusing on accuracy.

We also highlight the importance of transparency with respect to both the fairness and confidence of models, such that policymakers have the context they need to use models in ways that do not exacerbate inequities. While we frame these statistical and sociological approaches as they apply to machine-learned COVID-19 forecasting models, this method of developing and validating algorithms in a fairness-focused and transparent manner may be generalized to other forecasting approaches such as annual influenza modeling or forecasting of future pandemics, as well as to machine-learning endeavors across healthcare and social policy applications.

### Recognizing biases when selecting outputs

The decisions made around selecting model inputs have substantial implications for the potential biases that may be introduced. For example, algorithms designed to predict follow-up care by using healthcare spending have been shown to widen racial and ethnic disparities by failing to recognize that structural and financial inequities have led to disparities in healthcare spending independent of healthcare needs^20^. In this example, the selection of an improper output (healthcare spending instead of actual illness) led to the exacerbation of already inequitable health outcomes. Just as healthcare spending is reflective of structural inequities, many COVID-19 endpoints are deeply intertwined with structural inequities, and one must be careful when building COVID-19 forecasts to generate true forecasts and not merely extrapolate trends that represent baseline healthcare disparities.

One of the most important model inputs for pandemic forecasting has been clinical outcomes, especially as the clinical outcomes of COVID-19 themselves may be biased by underlying racial and ethnic disparities in healthcare access and quality. Pandemic forecasting presents a dual challenge of remaining fair and accurate while also providing useful and actionable policy insights. There is not always an overlap between the most reliable and most actionable variables, so it can be valuable to incorporate a mix of variables that provide sufficient quality of input data to make fair predictions while also providing useful outputs.

When building our COVID-19 model, we chose to include not only mortality, a relatively reliable and hard-to-miss variable, but also additional outputs for cases, hospitalizations, and ICU admissions. Details of the statistical methods underlying the Google-Harvard COVID-19 forecasting model can be found in a separate methods paper^31^. While each output offers value from a policy-planning standpoint, they differ substantially in their reliability. We recognized that case data are highly dependent on the number of tests performed in a community, and there is evidence that throughout the pandemic, Black and Hispanic communities have had substantially reduced access to testing relative to White communities^32^. If certain communities have less access to testing resources than others, they will appear to have fewer cases, and models may underestimate their future caseloads as well. Failure to account for this skewed data could then lead to a cycle in which models underestimate the healthcare needs of a low-resourced community, leading to further shifting of resources away from that community and exacerbating the undertesting and underestimation of cases already faced by that community. Hospitalization data may be more challenging to collect and aggregate from smaller and lower-resourced hospitals, leading to sparse input data that results in the least-accurate forecasts for the hospitals that serve the most vulnerable. Additionally, there are known racial and socioeconomic disparities in access to hospitals and critical care^33^, as well as in access to the health insurance coverage that enables individuals to seek out care^34^. These structural disparities may mask the true need for critical care in vulnerable populations and lead to embedded unfairness in hospitalization data. Thus, while cases and hospitalizations are certainly important to forecast for timely policy planning and intervention, models should include mechanisms to overemphasize higher-quality observations such as deaths while training.

### Including social determinants

Many socio-economic and related factors have been associated with increased rates of COVID-19 cases. In Massachusetts, for example, COVID-19 cases were highly associated with Hispanic ethnicity, but they were similarly associated with foreign-born immigrant status, greater household size, and share of foodservice workers^35^. Incorporating demographic and socioeconomic variables into forecasting models as predictors can help improve accuracy and allow models to recognize and account for inequities, yet there is concern that in some cases these variables may do more harm than good by amplifying structural inequities rather than adjusting for them. Concerns about this dichotomy are especially true with machine-learning techniques in which the role each variable plays in the ultimate predictions is largely obscured. Many demographic and socioeconomic variables are highly interrelated and may be correlated with confounding effects. Using such variables could cause a model to draw inaccurate explanatory insights and to rely directly on factors like race or gender even though these variables merely serve as proxies for other structural factors. This is perhaps best characterized by race, which is a social construct and largely representative of structural racism and inequities rather than innate differences between people and populations^10,36^. Additionally, much data on social determinants are often underreported or inaccurately reported, which could potentially mask underlying associations. When generalizing a model to new scenarios, there may then be an introduction of unfairness as the model continues to falsely rely on racial, gender, and other variables rather than on factors truly associated with the outcome of interest.

We recommend including socioeconomic factors in predictive models only if their effects on outputs are well-characterized by the scientific community and can be decoupled from the rest of data-driven learning. Specifically, we advise training models with and without each potentially problematic input variable and observing the effect on performance. The performance differences attributed to a specific factor could be quantified using analysis of variance tests that yield an f-statistic representing the importance of that factor. Those factors that do not significantly impact model performance can be excluded while those that do seem to have a major impact on performance should be further evaluated, drawing on social epidemiological and medical research, to validate that these observed associations are legitimate and not false or biased proxies for other more meaningful variables. If there are no scientific or sociological studies about the association, then further analysis should be employed to investigate the confounding effect, and modelers should clearly report any potentially confounding effects within their model. In the Google-Harvard forecasting model, we chose to avoid gender- and race-related variables as predictors in our forecasting model, though included age and population density as these were well understood to be directly linked to COVID-19 spread and severity. A more detailed explanation of how socioeconomic factors were incorporated into model transitions is available in the Google-Harvard Public Forecasting Model methods paper^31^. It is important, though, to use socioeconomic variables such as race and gender to stratify model outputs for fairness analyses, even if they are excluded as predictors during model training. A more detailed description of stratified fairness analyses is provided below.

### Choosing the appropriate geographic unit of analysis

In the US, it has been said, your zip code is more important than your genetic code. Given the legacy of segregation and structural inequity in the US, access to healthcare resources remains largely tied to geography^37^. With this clear association between geography and disparities in health outcomes, the geographic unit of analysis for pandemic forecasting models needs to be carefully considered by modelers and policymakers. Though state-level models provide more aggregated and higher-quality data inputs, they do not offer the jurisdictional granularity needed to guide policy or to accurately capture the uneven impact of the pandemic on areas with predominantly minority populations. This ecological fallacy is a well-studied problem, and while we highlight here some key considerations, there is considerable literature on small-area statistics and the intricacies of survey sampling^38–40^. Just as declining cases in New York caused a decline in national cases that hid exponential growth in Florida and Arizona in June of 2020, an example of Simpson’s paradox, building models at overly-aggregated geographies may conceal the critical heterogeneity that can guide policy action and reveal inequities. Capturing clustering and interaction within communities could perhaps best be accomplished by focusing on granular indicators like outbreaks in schools, but it should be noted that less-aggregated data can suffer from sparseness, lack of availability, and other issues of quality. When weighing the benefits and costs of different geographic units of analysis, we consider three fundamental challenges of more granular data:

(i) Data availability: our model, and other highly-accurate models, often use a wide range of data sources that correspond to case counts, healthcare availability, socioeconomic factors, mobility signals, non-pharmaceutical interventions, and other factors. These data sources are often not available beyond a certain granularity. Most states do report case counts at the county level but not at the zip-code level or other smaller areas. Most healthcare and socioeconomic data variables are reported at the county level as well, and mobility data is often limited to the county level due to privacy concerns. As such, we did not have the option to build our model at finer geographies than the county.
(ii) Informative content and forecastability: as we consider signals at higher granularity, their “forecastability” also decreases. Splitting signals into smaller pieces reduces the signal-to-noise ratio and, in other words, each occurrence becomes closer to a random process. Additionally, most variables become sparser at higher granularities which creates challenges for the supervision of end-to-end machine-learning models.
(iii) Interlocation dynamics: as the location granularity increases, interlocation dynamics, or the movement and mixing of people between geographic areas, become much more prominent. At the state level, the proportion of people spreading the disease to another state or using healthcare resources in another state is quite low. At the county level, there is much more inter-county mobility, disease spreading, and healthcare resource use. Beyond county-level granularity, interlocation dynamics can be very dominant and can challenge the independent, uniform mixing assumptions that most forecasting models, including the Google-Harvard model, rely on.

Based on the tradeoffs between accuracy and utility posed by different granularities of data, we ultimately chose to build both state- and county-level models in the US for our Google-Harvard COVID-19 Public Forecasting Model, and to use a prefecture-level model for Japan. Though models at the hospital-referral-region level or other aggregated geographies may have proved a valuable compromise, aggregating input variables like air quality, mobility, or other variables not well captured by a simple average limit the ability to build models at intermediate geographies. Recognizing that county-level data suffers from some of the issues of sparseness and inaccuracy mentioned above, we used data-imputation methods to fill in missing data and carefully conducted fairness analyses to help avoid introducing inaccuracy or unfairness into our predictions. Given previous work suggesting that data imputation itself can introduce bias^41^, we were especially careful in testing the fairness of our model and were transparent in reporting our imputation methods and the results of our fairness analyses so those seeking to use our model can recognize this potential source of bias and evaluate for themselves whether our model is sufficiently fair.

### Evaluating models through an equality lens

Whether comparing predictions to historical data or evaluating in real-time, assessment of model performance is a critical stage of development. While validation of accuracy is undoubtedly important, it is insufficient for a fairness-focused development process. Evaluation of model performance should be coupled with equality analyses in which forecasting models undergo subgroup analysis to verify that results are comparably accurate for each group and that there are no systematic biases in the model predictions. In order for the model to not introduce or reinforce any unfair decision-making, its forecasting accuracy should be similar across different subgroups when the population is partitioned based on race, socioeconomic status, sex, or other key factors that might highlight disparities. Potential approaches include ensuring that the mean absolute error and mean absolute percentage error are not systematically greater in key vulnerable populations. For example, in the Google-Harvard COVID-19 Public Forecasting Model, counties were divided into quartiles across a range of demographic variables, specifically age, gender, median income, race, and population density. The analysis then verified that the model had comparable errors across these groups, using mean absolute percentage error for comparisons^31^. We chose to conduct these fairness analyses at the county level, since distributions of demographic subpopulations tend to be fairly similar at the state level but show substantial variation at the county level. Though we ultimately found no major concerns in model performance across subgroups in the Google-Harvard COVID-19 Public Forecasting Model, we discuss below potential approaches to take when discovering that a model has unequal performance across subgroups. Conducting equality analyses can be helpful both for providing opportunities to fix those problems that may be fixable and to provide information on model limitations that cannot be fixed, allowing for greater transparency on the part of modelers and greater context available for policymakers seeking to use models in ways that do not worsen disparities. The effectiveness of these equality analyses can be further maximized by considering the consistency, directionality, and normalization of results.

#### Consistency across forecasting dates

Traditional mechanistic epidemic models include time points such as peak-time or epidemic doubling time, thereby capturing the dynamics of an epidemic^42^. However, given that COVID-19 is highly non-stationary and that parameters can change rapidly in response to behavior, testing, or interventions, equality analyses should be performed for varying relevant prediction dates and forecasting horizons in a given location. Assessing models across multiple time points is also valuable because phases of a pandemic can have varying disease dynamics across population subgroups. For example, the availability of vaccines initially to older Americans caused a larger reduction in mortality for elderly populations than for younger populations. Assessing a mortality prediction model at multiple time points can ensure that the model is making fair predictions of mortality for younger and older Americans both before and after the introduction of vaccines to specific groups. When selecting time points for equality analyses, it is also important to consider the intent of the model—to inform public health decisions or to illustrate long-term dynamics or counterfactual projections. Equality analysis should ideally be conducted both for short-term projections directed at changing behavior or policy and for longer-term projections, but the particular emphasis should be placed on analyses focused on time points most relevant to a model’s intended usage.

#### Accounting for the challenges of compartmental models

Traditional epidemiologic models for pandemics have relied on compartmental models such as the susceptible, exposed, infected, and recovered (SEIR) model. These compartments are then used to inform endpoints such as disease prevalence or death. While compartmental models are extremely powerful forecasting tools, they present an especially heightened concern with regard to bias and inequality. One risk of compartmental models is the assumption of similar behavior by subpopulations within each compartment. This assumption can be easily violated if social differences or structural disparities cause subpopulations to have different rates of infection or recovery. For example, individuals of low socioeconomic status in service or manufacturing jobs may be more likely to continue work on-site, increasing the likelihood of COVID-19 transmission compared to workers telecommuting from home. Though this assumption of uniform mixing is critical to achieving high forecasting accuracy with limited data and highly nonlinear dynamics, it provides a clear path for bias to be introduced into a model. When building compartmental models, it is important to understand the implications of this assumption and to select data and geographies that maximize the extent to which uniform mixing holds true. With the Google-Harvard Forecasting Model, we modeled compartmental transitions using machine learning based on numerous relevant input features, which can be helpful in mitigating the heterogeneity of compartments and mixing. Compartmental models also complicate efforts to ensure fairness through the nature of their compartmental structure, which relies on numerous interrelated and interacting variables. This interrelated structure is such that bias introduced in any compartment can be propagated and amplified into unequal and unfair model outputs across all compartments. Models should be built with the highest-quality data available and equality analyses should be conducted on multiple compartments and outputs to ensure that bias in internal compartments is not embedded into external predictions.

#### Normalizing to real-world magnitude and effect sizes

Since the prevalence of COVID-19 has varied markedly between communities, the raw errors of a predictive model can vary in ways that obscure its true fairness. Specifically, larger subgroups will have greater absolute errors even if a model is not biased against them. Failing to account for this could lead equality analyses to reflect the underlying sample size and distribution of data rather than the actual fairness or unfairness of the model. A model designed to predict cases in both White and Indigenous populations in the US, for example, would likely show a much greater mean absolute error in the White population, simply because there are many more White individuals than Indigenous individuals living in the US. Normalization to account for this imbalance in population size might reveal that in fact, the model makes less accurate predictions for the Indigenous population than for the White population. Similarly, a community with a higher incidence of COVID-19 may see greater absolute errors solely because a model is predicting larger numbers of cases than in a similar community with a smaller infection rate. This on its own should not be considered unfairness, and errors should therefore be normalized to real-world data. Normalizing also helps ensure that any unfairness in predictions between subgroups will not be obscured by varied community sizes or differing rates of COVID-19 between subgroups. For the Google-Harvard COVID-19 Public Forecasting model, we normalized errors to the cumulative deaths within a state or county when conducting equality analyses. While the scope of disparities during the COVID-19 pandemic is such that no variable is wholly untainted by bias and inequity, we felt deaths were the most concrete variable available and the least likely to be substantially undercounted in low-resourced settings.

### Remedying any unfairness identified in models

Proper equality analyses are vitally important to ensuring a deep understanding of any concerns of unfairness in a model as well as the potential sources of that unfairness. When models are found to perform differently across subgroups, steps must be taken to ensure that unfair models are not used by policymakers in ways that may inadvertently widen health disparities. While the specific steps taken to address an unfair model may depend on the details of a model’s sources, methods, and desired uses, we recommend three broad approaches to remedy unfairness in forecasting models. Modelers may need to experiment with multiple approaches to find a successful strategy.

#### Data analyses and modifications

The collection of underlying data needs to be closely examined. Bias could arise from the use of variables reflecting structural disparities, from the selection of overly-granular geography, or from the use of low-quality data sources. Individual data sources should be evaluated both qualitatively and via statistical analyses (e.g., quantifying the correlation between them and subgroup populations). Based on the results of this investigation, modelers can choose not to use problematic data sources, ideally replacing them with less biased data. The model can then be retrained, and equality analyses can be repeated to see if modifications to model inputs have helped alleviate the unfairness of the model.

#### Model training modifications

There is a wide literature on technical approaches to improve fairness in modeling, including methods such as adversarial debiasing, fair representation learning, and fairness-constrained optimization^43–45^. These methods can add an additional objective to model training that promotes fairness as well as accuracy. In general, these methods can cause a small decrease in model accuracy but a significant gain in fairness. After adjusting the training methodology of a model, it is important to conduct both traditional analyses of accuracy as well as equality analyses to ensure that the methodological change results in a reasonable and beneficial tradeoff between overall accuracy and subgroup fairness.

#### Transparent reporting of potential remaining fairness issues

If modifications to both the input data and model training methodology are insufficient to fully eliminate concerns about the fairness of a model, it is important for the users (policymakers, healthcare managers, etc.) to acknowledge and understand these concerns. Many models may suffer from some small but inevitable levels of unfairness while still offering valuable insight. Modelers should therefore clearly and explicitly report these fairness concerns alongside the results of the model. This reporting can be done both through explicit disclosures and through structuring predictions to highlight the level of confidence. In the Google-Harvard COVID-19 Public Forecast model, we used bootstrapping to estimate confidence intervals by sampling with replacement across prediction dates and locations. In addition, we chose to provide results as confidence intervals and with statistical significance tests rather than simply as point estimates, so that our accuracy and fairness across regions were clear and transparent. Policymakers armed with this knowledge can then use models to help guide decision-making while understanding the context around confidence and fairness and while taking action to mitigate the potential inequities that could be introduced if the model was fully trusted and used naively. Reporting the results of equality analyses can be valuable even in situations where no concerns are observed, as this can provide confidence to those considering the model and can increase the likelihood that models are used in actionable and valuable ways.

## Conclusion

In recognition of the potential harm of building and using pandemic forecasting models without explicit acknowledgment of structural inequity, one silver lining of the COVID-19 pandemic should be the opportunity and responsibility for forecast modelers to adopt an explicit framework around fairness and equity. Modelers should carefully consider the outputs they predict, the geographies for which they make their predictions, the social determinants they include as predictors, and the downstream implications of their predictions. They should also explicitly evaluate the fairness of their models before releasing them into the world and should be fully transparent so that others can understand the development and testing of their models. Of equal importance, policymakers must understand both the promises and the pitfalls of forecast results and should strive to use models in ways that promote equity and avoid perpetuating inequities. By deliberately working to improve the fairness of forecasting models, we can alleviate concerns, promote equity, and help forecasting models achieve their promise of providing a valuable policy tool to those seeking to respond to the COVID-19 pandemic and future healthcare emergencies.

### Reporting summary

Further information on research design is available in the Nature Research Reporting Summary linked to this article.

## Supplementary information

Reporting Summary Checklist

## Data Availability

Data sharing not applicable to this article as no datasets were generated or analyzed during the current study.
